# Repression of Invasion Genes and Decreased Invasion in a High-Level Fluoroquinolone-Resistant *Salmonella* Typhimurium Mutant

**DOI:** 10.1371/journal.pone.0008029

**Published:** 2009-11-25

**Authors:** Anna Fàbrega, Laurence du Merle, Chantal Le Bouguénec, M. Teresa Jiménez de Anta, Jordi Vila

**Affiliations:** 1 Department of Microbiology, Hospital Clínic, School of Medicine, University of Barcelona, Barcelona, Spain; 2 Institut Pasteur, Pathogénie Bactérienne des Muqueuses, Paris, France; Charité-Universitätsmedizin Berlin, Germany

## Abstract

**Background:**

Nalidixic acid resistance among *Salmonella* Typhimurium clinical isolates has steadily increased, whereas the level of ciprofloxacin resistance remains low. The main objective of this study was to characterize the fluoroquinolone resistance mechanisms acquired in a *S*. Typhimurium mutant selected with ciprofloxacin from a susceptible isolate and to investigate its invasion ability.

**Methodology/Principal Findings:**

Three different amino acid substitutions were detected in the quinolone target proteins of the resistant mutant (MIC of ciprofloxacin, 64 µg/ml): D87G and G81C in GyrA, and a novel mutation, E470K, in ParE. A protein analysis revealed an increased expression of AcrAB/TolC and decreased expression of OmpC. Sequencing of the *marRAB*, *soxRS*, *ramR* and *acrR* operons did not show any mutation and neither did their expression levels in a microarray analysis. A decreased percentage of invasion ability was detected when compared with the susceptible clinical isolate in a gentamicin protection assay. The microarray results revealed a decreased expression of genes which play a role during the invasion process, such as *hilA*, *invF* and the *flhDC* operon. Of note was the impaired growth detected in the resistant strain. A strain with a reverted phenotype (mainly concerning the resistance phenotype) was obtained from the resistant mutant.

**Conclusions/Significance:**

In conclusion, a possible link between fluoroquinolone resistance and decreased cell invasion ability may exist explaining the low prevalence of fluoroquinolone-resistant *S*. Typhimurium clinical isolates. The impaired growth may appear as a consequence of fluoroquinolone resistance acquisition and down-regulate the expression of the invasion genes.

## Introduction

*Salmonella enterica* is a Gram-negative facultative intracellular anaerobe of worldwide importance causing gastroenteritis in humans after ingestion of contaminated food or water. Serovars Enteritidis and Typhimurium are the most frequently isolated among the more than 2500 serovars characterized in *Salmonella enterica* [1], [2]. Upon colonization of the intestine by virulent strains, bacteria localize to the apical epithelium and induce invasion-associated virulence machinery [2]. Most of these virulence genes are organized within particular regions of the genome, termed pathogenicity islands, which are regulated by complex regulatory networks: the delicate balance of expression of many genes acting at the correct time in the correct location [3], [4]. Thus far, a total of five *Salmonella* pathogenicity islands (SPIs) have been described which are involved in causing disease by allowing invasion of eukaryotic cells as well as their survival and dissemination within the organism [3]. Furthermore, SPI-1 [5] and SPI-2 [6] have been reported to encode the specific machinery that delivers the effectors into the cytoplasm of the eukaryotic cells; these are the so-called type 3 secretion systems (T3SS) which play a central role in the *Salmonella*-host interaction [7].

Specific antimicrobial therapy is only indicated in the presence of positive signs of invasive disease, as symptoms usually resolve spontaneously. However, immunocompromised patients require treatment to prevent invasion [2], [8]. The most appropriate treatment includes fluoroquinolones, trimethoprim-sulfamethoxazole (TMP-SMZ), ampicillin, or third generation cephalosporins (ceftriaxone or cefixime). However, since resistance to ampicillin and TMP-SMZ is common, [1] representing ∼57% and ∼69% in 2004, respectively [8], use of a third-generation cephalosporins and quinolones seems to be a more reasonable choice when susceptibilities are unknown.

Nevertheless, quinolone resistance is an emerging problem not only in clinical strains isolated from humans but also in strains from livestock [9]. Over the last years several studies, including unpublished data from the Microbiology Service of our hospital, have been reported showing an increasing frequency of nalidixic acid-resistance (MIC>16 µg/mL) linked with a decreased ciprofloxacin susceptibility level (0.125 µg/mL) [10], [11]. In Europe, this percentage increased from 14% among *Salmonella* spp. clinical isolates in 2000, to 20% in 2004. However, ciprofloxacin resistance (MIC>1 µg/mL) is less frequent, remaining unchanged at around 0.8% [8], [10].

Although plasmid-mediated quinolone resistance has been described, the main mechanism of acquisition of fluoroquinolone resistance in *Salmonella* spp. has been attributed to chromosomal mutations, such as those characterized within the QRDRs (quinolone resistance-determining regions) of the target genes (the *gyrA* and *gyrB* genes encoding the A and B subunits of the DNA gyrase, respectively, and the *parC* and *parE* genes encoding the A and B subunits of the topoisomerase IV, respectively) and those affecting the accumulation of the antibiotic by decreasing its uptake as a consequence of a decrease in porin expression or by increasing the efflux of the drug related to an overexpression of efflux pump(s) [12]–[14]. AcrAB/TolC is the main efflux pump characterized which plays a key role in fluoroquinolone resistance and in conferring the MAR phenotype [15]–[18].

According to these clinical data, we hypothesized that fluoroquinolone resistance may appear concomitantly with a loss or decrease in expression of virulence factors, such as those that determine *Salmonella* invasion ability, leading to an impaired phenotype unable to adhere to or invade the epithelium *in vivo*, and consequently, meaning that these resistant strains would not be able to adhere to/invade the intestinal epithelia and therefore they could not be detected as a cause of human disease.

The main objective of this study was to investigate the possible relationship between quinolone resistance acquisition and expression of virulence factors. Furthermore, in depth characterization of the quinolone resistance mechanisms as well as the whole process of becoming a high-level resistant mutant were also a matter of concern.

## Results

### Characteristics of the Resistant Mutants: QRDR Mutations and Effect of Efflux Pump/s

A high-level ciprofloxacin resistant mutant (strain 50–64, MIC of 64 µg/mL) was obtained from a *Salmonella* Typhimurium clinical isolate which was ciprofloxacin susceptible (strain 50-wt, MIC of 0.012 µg/mL). In order to study the whole process of high-level fluoroquinolone resistance acquisition, intermediate mutants (50-0.007, 50-0.015, 50-0.03, 50-0.6, 50-0.25, 50-2 and 50-16) of this stepwise selection procedure were also included.

Analysis of mutations within the QRDRs of the *gyrA*, *gyrB*, *parC* and *parE* genes, as well as evaluation of the MICs of ciprofloxacin, norfloxacin and nalidixic acid were performed for each selected strain (Table 1). MICs were further determined in the presence of 20 µg/mL PAβN (Phenyl-Arginine-β-Naphthylamide), an efflux pump inhibitor. Sequencing results revealed that strain 50–64 had acquired three different amino acid changes. The first occurred in GyrA, D87G, of strain 50-0.06. The other two changes appeared at the same time in strain 50-16, G81C (GyrA) and a non-previously described mutation at the amino acid codon E470K (ParE).

**Table 1:** MIC determinations in the presence and absence of PAβN and mutations detected within the QRDRs.

Strain	MIC (µg/mL)a,b						Amino Acid Substitutionc,d				
	CIP		NOR		NAL		GyrA		GyrB	ParC	ParE
50-wt	0.012	(0.012)	0.094	(0.094)	4	(0.5)	---	---	---	---	---
50-0.007	0.012	(0.012)	0.094	(0.094)	4	(0.5)	---	---	---	---	---
50-0.015	0.032	(0.012)	0.19	(0.125)	8	(0.5)	---	---	---	---	---
50-0.03	0.064	(0.023)	0.5	(0.19)	24	(0.5)	---	---	---	---	---
50-0.06	0.38	(0.19)	3	(2)	256	(8)	---	D87G	---	---	---
50-0.25	0.38	(0.19)	3	(2)	256	(8)	---	D87G	---	---	---
50-2	1.5	(0.19)	12	(2)	256	(8)	---	D87G	---	---	---
50-16	32	(1)	256	(16)	4096	(32)	G81C	D87G	---	---	E470K
50-64	64	(1)	512	(16)	4096	(32)	G81C	D87G	---	---	E470K
50-rev	1.5	(1)	24	(16)	512	(32)	G81C	D87G	---	---	E470K

a CIP, ciprofloxacin; NOR, norfloxacin, NAL, nalidixic acid.

b Numbers in parenthesis represent the MICs determined in the presence of PAβN (20 µg/mL).

c ---, no mutation found.

d G, glycine; C, cysteine; D, aspartic acid; E, glutamic acid, K, lysine.

The resistance profile revealed that strain 50–64 had a 5333-, 5446- and 1024-fold increase in the MICs of ciprofloxacin, norfloxacin and nalidixic acid, respectively, in comparison to strain 50-wt (Table 1). Upon the addition of PAβN, only an 83.3-, 170- and 64-fold increase in the MIC of the same antibiotics was detected when making the same comparison, suggesting that this partial increment in the resistance phenotype may be attributed to the QRDR mutations. In addition, these results also indicate that the remaining increment in resistance (64-, 32- and 128-fold) until the final MIC values are reached may be attributed to at least one efflux pump susceptible to this inhibitor.

When taking into account both results, QRDR mutations and MICs in the presence of PAβN at the same time, a good-correlation was observed between the largest increments in the MICs of quinolones between consecutive mutants and the acquisition of the target gene mutations: strain 50-0.06 (D87G in GyrA) showed a 8.3-, 10.5- and 16-fold increase in ciprofloxacin, norfloxacin and nalidixic acid resistances, respectively, in comparison with strain 50-0.03, the previous mutant selected; and strain 50-16 (G81C in GyrA and E470K in ParE) showed a 5.3-, 8- and 4-fold increase in the same MICs in comparison with 50-2, the previous mutant selected.

On comparing the results obtained from the MICs performed with and without PAβN, 6 different steps may be taken into consideration: i) the first step (strain 50-0.015) appears prior to the acquisition of any QRDR mutation, when the ciprofloxacin concentration in the media is similar to the MIC of the initial strain (0.015 µg/mL), and represents a small increase in the MICs of the three quinolones tested (1.5- to 3-fold). ii) The second step (strain 50-0.03) mainly represents a further increase in the MIC of nalidixic acid (3-fold). iii) The third step (strain 50-0.06) is characterized by the acquisition of the first target gene mutation in the *gyrA* gene (D87G) concomitantly with a large increment of the three MICs (8- to 16-fold in the presence of PAβN). No sign of a PAβN-susceptible mechanism is detected at this point. iv) The fourth step (strain 50-2) only affects the MICs of ciprofloxacin and norfloxacin with a 4-fold increase. v) The fifth step (strain 50-16) combines, on one hand, two QRDR mutations (in the *gyrA* (G81C) and *parE* (E470K) genes) that can be associated with an increment of about 4- to 8-fold concerning all the quinolones in the presence of PAβN. However, with these data, it is not possible to elucidate the partial contribution of each mutation. On the other hand, another 2.7- to 4-fold increase in the MIC of the three types of quinolones used can be attributed to a mechanism susceptible to the presence of PAβN. Finally, vi) the sixth step (strain 50–64) shows a 2-fold increase enhancing the MICs of ciprofloxacin and norfloxacin reaching the maximum values of resistance.

### The Quinolone Resistance Phenotype Can Be Partially Reverted in the Absence of the Antibiotic

In addition to the fluoroquinolone resistant mutants selected in the presence of ciprofloxacin, strain 50–64 was further examined to evaluate if a total or partial reversion of the resistance phenotype could occur under non-selective conditions. Strain 50–64 was grown in the absence of ciprofloxacin 42 consecutive days and the resulting strain, 50-rev, was characterized. Although this strain had preserved the same QRDR mutations acquired previously during the stepwise process, it showed a 43-, 21- and 8-fold decrease in the MICs of ciprofloxacin, norfloxacin and nalidixic acid, respectively, in comparison with strain 50–64; whereas no significant change could be detected in the MICs in the presence of PAβN (Table 1).

### Fluoroquinolone Resistance Associated with the Multiple Antibiotic Resistance (MAR) Phenotype

Strain 50–64 was analyzed to determine if a MAR phenotype emerged during the quinolone resistance acquisition process. The MICs of chloramphenicol, tetracycline, β-lactams (amoxicillin, ceftriaxone and cefoxitin), erythromycin, kanamycin and trimethoprim were assessed and are shown in Table 2. All antibiotics showed a significant increase in their MICs when comparing strain 50–64 with 50-wt with the exception of kanamycin. Futhermore, these increasing values concerning the MAR phenotype, could revert totally or partially to the wild-type level in strain 50-rev (Table 2).

**Table 2:** Characterization of the MAR phenotype.

Strain	MIC (µg/mL) a							
	CHL	TET	AMX	CRO	FOX	ERY	KAN	TMP
50-wt	3	3	6	0.064	3	32	1	0.25
50-64	>256	32	>256	1	>256	>256	1.5	6
50-rev	4	2	8	0.19	6	96	2	1

a CHL chloramphenicol, TET tetracycline, AMX amoxicillin, CRO ceftriaxone, FOX cefoxitin, ERY erythromycin, KAN kanamycin, TMP trimethoprim.

### Sequencing Analysis of Transcriptional Factors Leading to the MAR Phenotype

**Figure 1:**
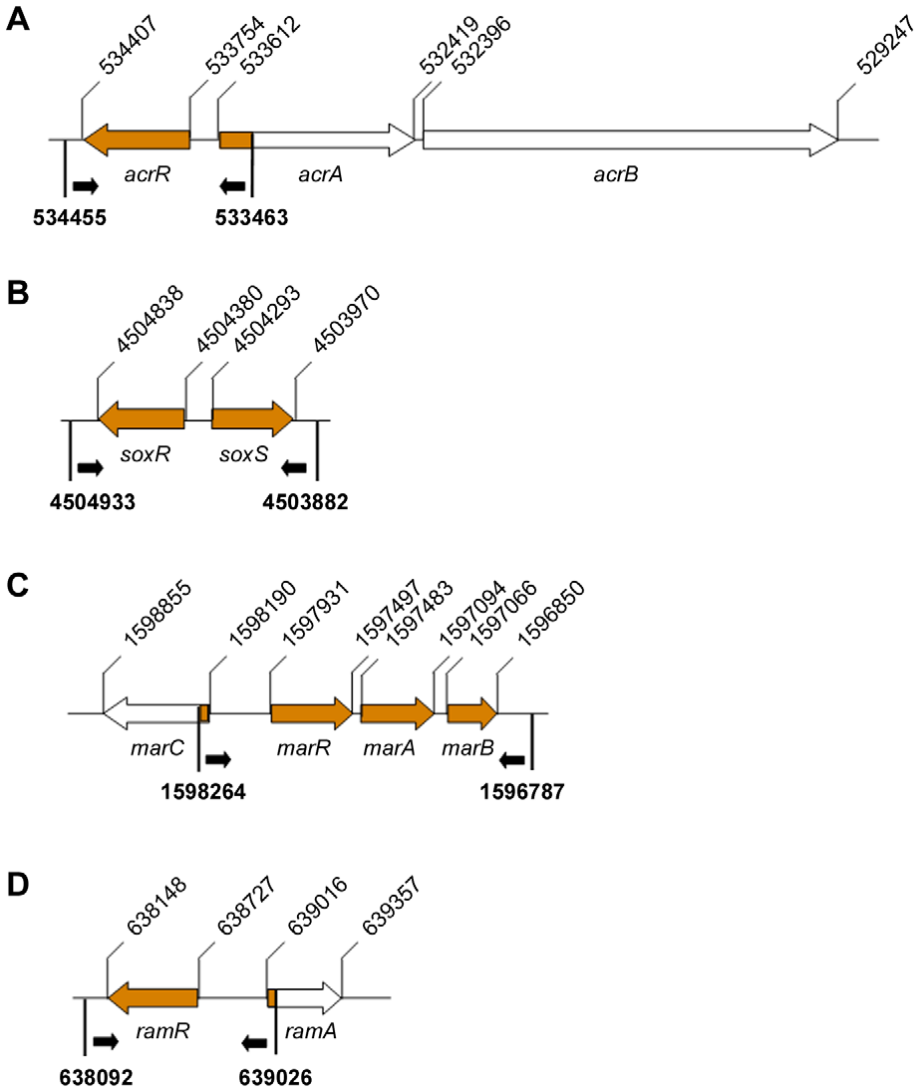
Sequencing map of the known efflux regulators. Schematic representation of the sequences analyzed for detection of mutations within the regulatory loci operons: *acrR* (A), *soxRS* (B), *marRAB* (C) and *ramR* (D). Open arrows represent the genes of each operon, dark background indicates the fragments analyzed. Small black arrows indicate the orientation of the primers. Upper numbers indicate the nucleotide positions of each gene according to the S. Typhimurium LT2 GenBank Accession No. NC_003197, whereas bold-face numbers indicate the location of the primers.

Since the MAR phenotype agrees with the substrate profile of AcrAB [15], [19], sequencing of the regulatory loci (*acrR*, *soxRS*, *marRAB* and *ramR*) reported to regulate AcrAB expression, as well as their promoters, was performed in order to detect any possible mutation that could justify the MAR phenotype (Figure 1). However, the sequencing results showed that there was no nucleotide substitution in any of the sequences evaluated.

### Bacterial Growth

**Figure 2:**
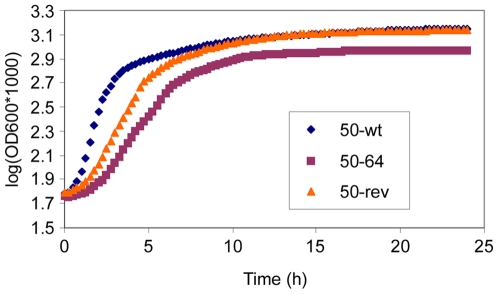
Bacterial growth. Bacterial growth curves of strains 50-wt, 50–64 and 50-rev are compared. Measures were taken every 15 minutes for 24 hours from four independent data for each strain. The results are expressed in a semilogarithmic plot.

In order to compare the fitness of strains 50-wt, 50–64 and 50-rev, growth was measured for each strain. The OD_620_ was measured every 15 minutes for 24 hours and the results are shown in Figure 2. In terms of growth rate (μ = (lnN−lnN_0_)/(t−t_0_)), a significant difference between strains 50-wt and 50–64 (*P*<0.05) was of note with the latter clearly showing a much longer lag-phase until the OD significantly increases. Strain 50-rev showed a lag-phase more similar to that of 50–64 during the first two hours as well as an intermediate growth rate which was still significantly different from that of 50-wt (*P*<0.05) and 50–64 (*P*<0.05). However, strain 50-rev eventually reached the same stationary values than those of 50-wt.

### Invasion Assays

**Figure 3:**
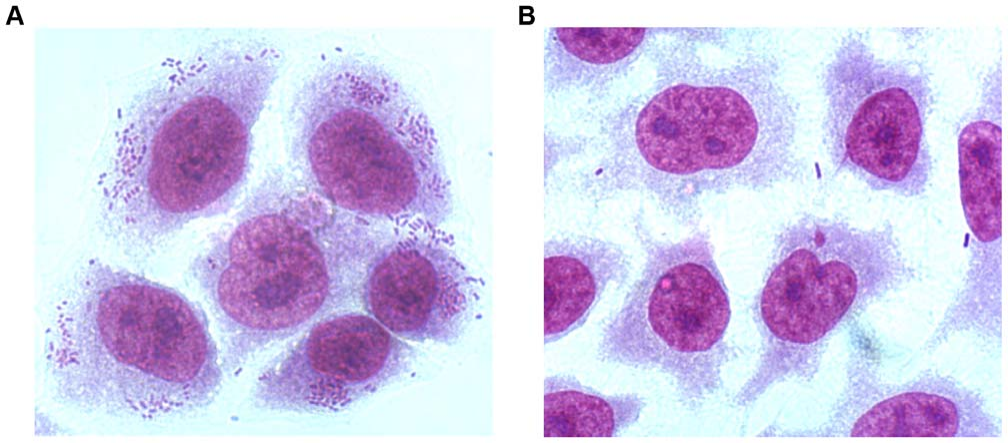
Cell invasion. Optical microscope images of OHIO cells after an infection of 2.5 hours with strains 50-wt (A) and 50–64 (B). Cells were stained with Giemsa stain.

A gentamicin protection assay was performed to determine if there had been any change in the invasion ability of the high-level resistant mutant (50–64) in comparison with the susceptible isolate, 50-wt. In addition, 50-rev was also tested. Results are expressed as a percentage of the number of invasive bacteria with respect to the total number of bacteria present in the initial inoculum. A clear decrease was observed in the number of bacteria interacting with the epithelial cells in strain 50–64 with respect to 50-wt when comparing the images shown in Figure 3. The percentage of invasion significantly decreased from 11.1% for strain 50-wt to 0.2% for strain 50–64 (*P*<0.05). However, strain 50-rev only showed a percentage of 0.7%, a very small increase compared to that of the resistant mutant which was not sufficient to be considered as a significant reversion (*P*>0.05). Results are shown in Table 3.

**Table 3:** Percentage of invasion ability.

Strain	% Invasion^a^	
	Mean	±SD^b^
50-wt	11,1	6,2
50-64	0,2	0,1
50-rev	0,7	0,5

a Bacteria surviving treatment with gentamicin as a percentage of total bacteria.

b SD, Standard Deviation of n = 3 independent assays.

### Microarrays I: Evaluating the Resistance Phenotype

A microarray analysis was carried out in order to compare the differential expression of the genes leading to the observed phenotype. Two distinct analyses were performed: the first was a comparison between the levels of expression of strain 50–64 related to the basal expression of 50-wt. The aim was to determine the putative genes leading to the high-level fluoroquinolone resistance phenotype but also to justify the decreased percentage of invasion ability. The second analysis was a comparison between the levels of expression of strain 50-rev related to the expression of 50–64 to detect the genes that could have reverted towards a wild-type condition. The data from each gene was provided from two independent experiments. Positive values refer to genes that are up-regulated, whereas negative values refer to repression of expression (Table 4). Some genes were found to have an impaired expression linked with their known function in conferring quinolone resistance. The first analysis showed an increased expression of *acrAB* (>2-fold) whereas *tolC* increased but to a lesser extent (1.83/1.06). The second analysis showed a decrease in the same mRNA transcripts suggesting a total or partial reversion. The microarray results did not show any altered expression of any known transcriptional regulator of AcrAB (*acrR*, *marA*, *soxS* neither *ramA*) (data not shown). This result corroborates the fact that no mutation was found within their regulatory loci as previously mentioned. In addition, a decreased expression of *ompC* in the resistant strain was detected in the first analysis followed by higher levels of expression in the second. The values for each gene are shown in Table 4.

**Table 4:** The most significant genes detected in the microarrays results.

			Microarray analysesa,b			
Phenotype and gene		Product	50–64 vs 50-wt^c^		50-rev vs 50–64^d^	
Resistance phenotype						
	*acrA*	acridine efflux pump	2.71	2.80	−2.51	−1.63
	*acrB*	acridine efflux pump	2.11	2.51	−2.25	−1.88
	*tolC*	outer membrane channel precursor protein	1.83	1.06	−1.35	−1.07
	*ompC*	outer membrane porin protein C	−1.98	−1.47	2.34	2.55
Invasion phenotype						
SPI-1	*hilA*	invasion protein transcriptional activator	−8.54	−4.88	1.95	1.57
	*invF*	invasion regulatory protein	−8.04	−4.42	1.41	1.12
	*hilD*	invasion regulatory protein	−2.42	−1.25	1.22	1.19
	*hilC*	invasion regulatory protein	−5.51	−3.01	1.89	1.63
SPI-4	*siiB*	putative methyl-accepting chemotaxis protein	−3.76	−2.06	1.43	1.34
	*siiC*	putative ABC exporter outer membrane component	−6.57	−3.14	1.25	1.73
	*siiD*	membrane permease; HlyD secretion protein	−5.56	−6.40	1.88	1.87
	*siiE*	putative inner membrane protein	−1.09	−1.25	1.41	1.05
	*siiF*	putative ABC-type bacteriocin/lantibiotic exporter	1.12	1.04	1.41	1.04
SPI-5	*sigD*	sopB | secreted effector protein	−6.10	−3.47	1.69	1.55
	*sigE*	pipC | pathogenicity island-encoded protein C	−2.83	−1.60	1.37	1.07
Flagella	*flhD*	transcriptional activator FlhD	−2.95	−1.63	1.73	2.07
	*flhC*	flagellar transcriptional activator	−4.19	−3.41	2.81	2.07

a each microarray analysis is provided with two independent data.

b + indicates up-regulation of the genes, − indicates down-regulation.

c gene expression of 50.64 relative to expression of 50.wt.

d gene expression of 50.rev relative to expression of 50.6.

### Microarrays II: Evaluating the Invasion Phenotype

As far as the invasion phenotype is concerned, many genes showed a decreased expression in strain 50–64 in the first analysis of the microarrays, and, in addition, most of these showed an increase, although to a lesser extent, in strain 50-rev in the second analysis. These affected genes included several operons whose function has been shown to be important during the invasion process. In the first analysis, all genes encoded within the SPI-1 showed a decreased expression, including the structural genes (those encoding the T3SS-1) and primary effectors, encoded in the *prg/org*, *inv/spa* and *sic/sip* operons, as well as the transcriptional activators, such as *hilA*, *hilC*, *hilD* and *invF*. In the second analysis, a partial increase in their expression was detected (Table 4).

Alternatively, genes belonging to the other SPIs were analyzed to detect if they could also show an impaired expression. Genes encoded within SPI-2 [20] and SPI-3 [21] did not show any significant change in their expression (data not shown). However, the same was not true concerning the genes encoded within SPI-4 and SPI-5. Intriguingly, when analyzing the six-gene operon encoded in SPI-4 (*siiABCDEF*) [22], only the *siiB*, *siiC* and *siiD* genes showed a significantly decreased expression (2- to 7-fold) (the *siiA* gene could not be detected among microarray data, whereas *siiE* and *siiF* did not show any significant change). In addition, the main operon described in SPI-5, *sigDE* [23], also showed a reduced expression, mainly *sigD*, in 50–64 in comparison with the susceptible isolate (Table 4). Furthermore, most of the genes belonging to the operons involved in the synthesis and assembly of the flagellar apparatus as well as chemotaxis: *flg*, *flj*, *fli*, *mot* and *che* (Table S1), including the regulatory genes *flhDC* (Table 4), consistently showed negative values in the first analysis despite being affected to a different extent. Additionally, positive values were detected for these genes in the second analysis, albeit no clear or significant reversion could be concluded.

### mRNA Analyses by RT-PCR

**Figure 4:**
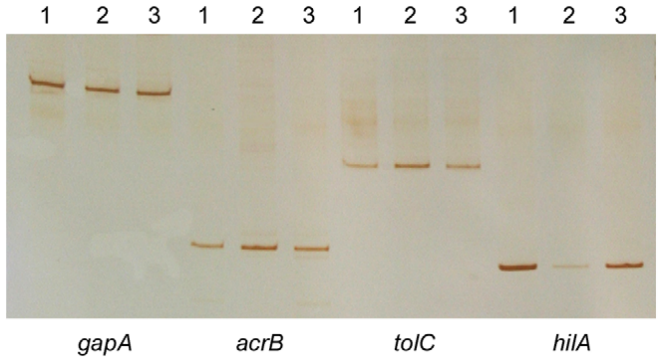
Gene expression analysis by RT-PCR. The RT-PCR assay performed to detect the levels of expression of the *acrB*, *tolC* and *hilA* genes. The *gapA* gene was the internal control used to detect if similar amounts of RNA were added for each strain assays. Lane 1, strain 50-wt; lane 2, strain 50–64, lane 3, strain 50-rev.

The most important genes with a crucial putative role in the final phenotype were selected to confirm their expression by RT-PCR (Figure 4). Assays were focused on *acrB*, *tolC* and *hilA*, as the most significant genes to corroborate results from microarray analyses. *acrB* as well as *tolC* showed an increase in strain 50–64 in comparison with 50-wt, whereas in 50-rev they decreased to almost the same levels of expression as 50-wt. By contrast, *hilA* showed a substantial decrease in strain 50–64 in comparison with 50-wt, which partially reverted in 50-rev.

### Protein Analyses by SDS-PAGE and Western Blotting

**Figure 5:**
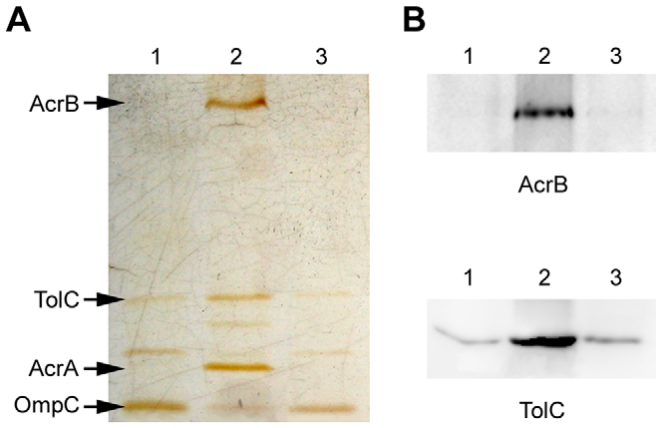
Protein analysis by SDS-PAGE and Western blot. Protein analysis was performed using cell envelope protein extracts loaded in a 12%. SDS-PAGE (A) and Western blot with antibodies against AcrB and TolC (B). Arrows indicate the specified proteins. Lane 1, strain 50-wt; lane 2, strain 50–64, lane 3, strain 50-rev.

A cell envelope protein extract was obtained from strains 50-wt, 50–64 and 50-rev and a sample of each was run in a SDS-PAGE (Figure 5). The resulting gel confirmed the same expression pattern observed for *acrB*, *acrA*, *tolC* and *ompC* mRNAs (results obtained from both RT-PCR and microarrays analyses). AcrB, AcrA and TolC proteins showed an increased expression in strain 50–64 in comparison with 50-wt, whereas they showed decreased levels in 50-rev reaching similar levels to that of 50-wt. In addition, an inverted effect could be detected for OmpC, showing a decreased expression in the resistant strain followed by a consecutive increase in 50-rev.

Furthermore, Western blot detection was performed using antibodies from rabbit against AcrB and TolC. The same results as those obtained above were corroborated as is shown in Figure 5.

## Discussion

The main purpose of this study has focused on understanding if, concurrent with the acquisition of fluoroquinolone resistance, there is a loss or repression of virulence factors, e.g. invasion proteins. This may explain the clinical scenario in which no increase in the resistance of Salmonella spp. to ciprofloxacin is observed whereas resistance to nalidixic has been steadily increasing. The first objective was to characterize the molecular mechanisms of fluoroquinolone resistance in a *Salmonella* Typhimurium mutant (strain 50–64, MIC of ciprofloxacin of 64 µg/mL) obtained *in vitro* from a highly susceptible clinical isolate (strain 50-wt, MIC of ciprofloxacin of 0.012 µg/mL) at increasing concentrations of ciprofloxacin. Intermediate mutants selected during the resistance stepwise process were also studied.

Comparative study between these strains revealed the acquisition of three QRDR amino acid changes. The first (A87G in GyrA) was found in strain 50-0.06. The other two mutations (G81C in GyrA and E470K in ParE) were simultaneously acquired in strain 50-16. Despite mutations in the *gyrA* and *parC* genes being the most commonly found and well-characterized in conferring quinolone resistance, mutations in the *gyrB* and *parE* genes have also been described, although their contribution, if any, to the resistance phenotype seems to be lesser [24]–[27]. Here a novel mutation in the *parE* gene is described. Although with the information reported in this study it is not possible to elucidate the contribution of this mutation to the resistance phenotype, it may be important to include not only the *gyrB* but also the *parE* genes in routine sequencing in order to clarify the possible role of these secondary mutations.

The MICs of quinolones were assessed in the presence of PAβN and showed evidence of efflux contribution to the resistance phenotype, ranging from 2.7-fold in strain 50-0.015, to 64-fold in strain 50–64, concerning ciprofloxacin resistance. Further experiments, such as RT-PCR and protein analyses, revealed the overexpression of AcrAB/TolC in 50–64. In addition, MICs of other unrelated drugs, such as β-lactams, chloramphenicol, tetracycline, erythromycin and trimethoprim, were evaluated and showed a significant increase in 50–64, whereas the resistance to kanamycin remained unchanged. These results likely encourage the involvement of AcrAB in the resistance phenotype since this substrate specificity matches that described for AcrAB/TolC [15], [19].

In addition to the resistant mutant, it was possible to obtain a strain with a reverted phenotype, strain 50-rev, from strain 50–64 which maintained all the QRDR mutations acquired previously but showed a significant decrease not only in the resistance levels to the quinolones tested but also to the other unrelated drugs. Concomitantly, the decrease in AcrAB/TolC expression that was detected in this strain is noteworthy. To date, this is the first report showing a partial reversion of the MAR phenotype acquired *in vitro* (including the high-level of fluoroquinolone resistance). These results suggest that a major part of this PAβN-susceptible mechanism may revert towards a wild-type condition in the absence of the selective pressure.

Several studies have reported that, in *Salmonella* spp., the first and essential step towards the resistance phenotype is the acquisition of mutations that gives rise to an increased efflux, mainly due to AcrAB overexpression, whereas mutations in the QRDRs represent the second step as well as other mutations enhancing the efflux activity [16], [17], [28]. The resistance phenotype observed in this stepwise process appears as a consequence of progressive increments during the whole procedure, suggesting that mutations are acquired at multiple steps. Therefore, we propose that six different steps occurred on selection with ciprofloxacin. Accordingly, the first step would be attributed to the implication of an efflux pump, whereas target gene mutations as well as enhanced efflux activity would be acquired in the following steps.

AcrAB/TolC overexpression has been reported to increase the efflux of the three quinolones tested in this study (nalidixic acid, norfloxacin and ciprofloxacin) [16], [29]. However, each of these steps in which efflux seems to play a role, does not affect all quinolones in a similar way, as different combinations can be detected. In agreement with these results, we suggest the implication of other efflux mechanisms, apart from AcrAB/TolC, which may each be related to impair quinolone susceptibilities in a particular way (either nalidixic acid by itself or a combination of ciprofloxacin and norfloxacin). This suggestion means that hitherto unknown mechanisms play different roles in the process of quinolone resistance acquisition. Nevertheless, this is not the first time that evidence of implication of other efflux pumps, apart from AcrAB, have been presented [16], [17], [27]. It has been clearly demonstrated that a reduced or lack of expression of OmpF is involved in conferring quinolone resistance [18]. Furthermore, several studies performed in *E. coli* also associate a decreased expression of OmpC with increased resistance to fluoroquinolones [30], [31]; while other studies performed in *S.* Typhimurium link its decreased expression with resistance to β-lactams [32], [33]. In this study a decreased OmpC expression was detected in the resistant mutant. These mechanisms, altogether in combination with AcrAB/TolC, are likely the explanation for the final phenotype oberserved. Furthermore, the regulatory protein/s responsible for the overexpression of these efflux pumps, after showing no implication of MarA, SoxS and RamA, remain unknown. However, they also seem to show an impaired expression capable of reverting in the absence of selective pressure.

The second objective was focused on the characterization of the invasion phenotype. In order to evaluate the percentage of the invasion, strains 50-wt, 50–64 and 50-rev were selected to perform a gentamicin protection assay. The results showed that there was a significant decrease: from 11.1% in strain 50-wt down to 0.2% in strain 50–64. However, strain 50-rev still showed a low percentage (0.7%), meaning that no significant reversion regarding this phenotype could be concluded. These results are in agreement with those of the microarrays and RT-PCRs presented above, in which a significant loss of expression of the operons encoded in SPI-1, SPI-4 and SPI-5 has been shown in strain 50–64, besides the slighter repression of those genes encoding flagellar assembly and function, motility and chemotaxis (*flg*, *flj*, *fli*, *flh*, *mot* and *che*). Of note was the impaired expression of the key regulators, HilA and the *flhDC* operon, respectively. In addition, the lack of a total recovery of the expression of all these genes in strain 50-rev is noteworthy and does not allow to consider a significant reversion either. This full set of genes participates in the first stage of disease to mediate efficient intestinal colonization and pathogenesis. Thus a general regulation has been suggested in order to synchronize their expression [4], [34]–[37]. These results agree with the presence of a general regulation since they are down-regulated in strain 50–64 and a partial increased expression is detected in strain 50-rev.

Expression of SPI-1 genes, particularly the main regulator *hilA*, is extremely coordinated by many environmental and global regulatory signals [38], [39]. Thus any suboptimal factor, including growth rate, results in repression of the expression of *hilA* [38], [40] and the *flhDC* operon [41]. A link has previously been proposed between reduced DNA supercoiling (due to the presence of DNA gyrase inhibitors) and regulation of gene expression [42], [43], such as the *proU* operon which encodes a glycine betaine transport system [44] and invasion genes such as *invA*, encoded within the SPI-1 [45]. An initial hypothesis to justify the link between fluoroquinolone resistance and decreased invasion ability suggested that the mutations acquired in the *gyrA* gene may be responsible for a reduced superhelicity causing a repression of these genes. However, the microarray results did not show any change in the expression of the *proU* operon. Furthermore, it has been described that an *Escherichia coli* strain with a mutation in the *gyrA* gene can still be motile in standard conditions of growth and even in the presence of low concentrations of a DNA gyrase inhibitor. Nevertheless, growth was also impaired under the same conditions that alter motility [46].

More recently, it has been reported that ciprofloxacin-resistant strains, both clinical isolates and *in vitro* mutants obtained from susceptible clinical isolates, showed a decrease in mRNA expression of *invA* and *avrA* genes (the only two SPI-1 genes tested) in addition to a decrease in cell invasion ability. They also suggested the possibility that mutations in *gyrA* may be the cause of the phenotype. Nevertheless, they observed a decreased growth rate in ciprofloxacin-resistant strains (MIC of ciprofloxacin ≥4 µg/mL) [47]. This particular phenotype was also reported in a previous study [28] and it was linked to two fluoroquinolone-resistant mutants obtained *in vitro* (MICs of ciprofloxacin of 8 and 16 µg/mL). In this study we report a significantly decreased growth rate in strain 50–64 in comparison to 50-wt. The reverted strain, 50-rev, showed an impaired growth rate with a longer lag-phase, more similar to that of 50–64, although it eventually reached the stationary values of the wild-type strain. Furthermore, the motility was tested for 50-wt, 50–64 and 50-rev and the results showed a significant decrease observed in 50–64 which was not able to revert to the basal motility of 50-wt in 50-rev, being more similar to that of 50–64 (data not shown).

According to this information and the results presented here, the most suitable hypothesis found to justify the coexistence of both phenotypes, fluoroquinolone resistance and decreased invasion ability, is that exposure to quinolones leading to a high-level of resistance may alter the growth rate, and it may be the connecting factor triggering the coordinated repression of the genes implicated in the invasion phenotype since the optimal environmental conditions for the expression of these gene is lost, e.g. in 50–64 and 50-rev during the first hours of growth. Next experiments will be focused on a better understanding of this hypothesis. Furthermore, based on the extensive microarray analyses results, in depth characterization of the molecular mechanisms leading to the fluoroquinolone resistance phenotype, such as hitherto efflux pumps and the regulators that govern their expression as well as expression of AcrAB, will be studied and characterized.

## Materials and Methods

### Bacterial Strains and Selection of Resistant Mutants

Strain 50-wt is a *Salmonella enterica* serovar Typhimurium clinical isolate recovered from a stool sample in the Department of Clinical Microbiology in the Hospital Clinic of Barcelona, Spain. A ciprofloxacin resistant mutant, strain 50–64, was obtained from 50-wt in a multi-step selecting process in the presence of ciprofloxacin. Strains were grown at 37°C on MacConkey plates. Ciprofloxacin (Fluka, Steinheim, Germany) was only present during the selection procedures, starting at 0.007 µg/mL (half of the MIC for 50-wt) and increasing 2-fold each step, until reaching a maximum concentration of 64 µg/mL. Single colonies were selected at each step to be grown at the consecutive ciprofloxacin concentration and simultaneously a sample was frozen and named according to the ciprofloxacin concentration of selection (e.g., strain 50-0.007 was selected at a ciprofloxacin concentration of 0.007 µg/mL). Certain intermediate mutants (50-0.007, 50-0.015, 50-0.03, 50-0.06, 50-0.25, 50-2, 50-16) were chosen during the multi-step sequential process. Furthermore, a reverted strain, 50-rev, was selected from 50–64 by growth of single colonies on MacConkey plates in the absence of ciprofloxacin after 42 consecutive steps.

### Susceptibility Testing

MICs of ciprofloxacin, norfloxacin, nalidixic acid, chloramphenicol, tetracycline, amoxicillin, erythromycin, kanamycin, trimethoprim, ceftriaxone and cefoxitin were determined by Etest (AB Biodisk, Solna, Sweden) according to the manufacturer's recommendations. The broth microdilution method was used to evaluate the MICs of ciprofloxacin, norfloxacin and nalidixic acid when maximum Etest values were reached. MICs were determined according to CLSI guidelines [48]. MICs of quinolones were also determined in the presence of 20 µg/mL of PAβN (Sigma-Aldrich, St Louis, MD, USA) in MH plates.

### Detection of Mutations in the Genes Encoding Quinolone Protein Targets and Regulatory Loci

Amplification of the QRDRs of *gyrA*, *gyrB*, *parC*, and *parE*, as well as the *soxRS*, *marRAB*, *acrR* and *ramR* regulatory loci (as it is already known, the transcriptional regulators SoxS, MarA and RamR exert a positive effect on AcrAB/TolC expression, whereas AcrR is the local repressor) was performed using the corresponding primers listed in Table 5. PCR was performed in 50 µl of 1x GoTaq Flexi Buffer with 1.5 mM MgCl_2_, 1.5 U of *Taq* enzyme (Promega, Madison, WI, USA), 0.2 mM each deoxynucleoside triphosphate (Invitrogen, Carlsbad, CA, USA) and 25 pmol each primer (Isogen, De Meern, The Netherlands), using the following temperature profiles: incubation at 94°C for 2 min; followed by 94°C for 30 s, 55–62°C for 30–120 s, and 72°C for 45 s for 30 cycles; with a final extension step of 72°C for 5 min. The appropriate annealing temperature is detailed in Table 5. The duration of the extension was 30 s for QRDR amplification, being 2 min for analyzing the regulatory loci. The PCR products were loaded in a 1.5% agarose gel, purified using Wizard SV gel and PCR clean-up system (Promega, Madison, WI, USA), and sent to Macrogen Inc (Seoul, Korea) for sequencing to allow comparison with wild-type sequences.

**Table 5:** List of all primers used in this study.

Primer use and gene	Primer	Sequence 5′-3′	Product size	Temperature (°C)	n° of cycles	Reference
QRDR						
* gyrA*	gyrA.Sal1	AAATCTGCCCGTGTCGTTGGT	344 pb	58°C	30	this study
	gyrA.Sal2	GCCATACCTACTGCGATACC				
* gyrB*	SgyrB.1	GAATACCTGCTGGAAAACCCAT	446 pb	57°C	30	this study
	SgyrB.2	CGGATGTGCGAGCCGTCGACGTCCGC				
* parC*	parC.Sal1	AAGCCGGTACAGCGCCGCATC	395 pb	57°C	30	this study
	parC.Sal2	GTGGTGCCGTTCAGCAGG				
* parE*	SparE.1	CCTGCGGCCCGGCGTTGCCGGGG	465 pb	62°C	30	this study
	SparE.2	CGCCCGCCTTCTCTTCTTCCGTCAGCGCG				
Regulatory genes						
* soxRS*	Ssox.1	GGCACTTTGCGAAGGCGTTACCA	1052 pb	54°C	30	this study
	Ssox.2	GGGATAGAGCGAAAGACAA				
* marRAB*	Smar.1	AGCGGCGGACTTGTCATAGC	1476 pb	58°C	30	[51]
	Smar.2	ACGGTGGTTAGCGGATTGGC				
* acrR-acrA*	Sacr.1	CAGTGGTTCCGTTTTTAGTG	1012 pb	58°C	30	[51]
	Sacr.2	ACAGAATAGCGACACAGAAA				
* ramR*	SramR.1	CGTGTCGATAACCTGAGCGG	933 pb	62°C	30	[52]
	SramR.2	AAGGCAGTTCCAGCGCAAAG				
RT-PCR						
* gapA*	SgapA.RT1	GTATCAACGGTTTTGGCCG	610 pb	58°C	16	this study
	SgapA.RT2	GTAGAGGACGGGATGATGTTCT				
* acrB*	SacrB.RT1	GCGCGACGTTGATTCCGACTATTG	375 pb	58°C	19	this study
	SacrB.RT2	GGATCAGCGCGACCAGCACCGACA				
* tolC*	StolC.RT1	TACGCGTTGATGCTGCTGATGGAG	515 pb	58°C	18	this study
	StolC.RT2	ACCGCCCGCAACAACCTGGATA				
* hilA*	ShilA.RT1	CGCCGGCGAGATTGTGAGTAAAAA	356 pb	58°C	22	this study
	ShilA.RT2	TGCGGCAGTTCTTCGTAATGGTCA				

### Bacterial Growth

Overnight bacterial cultures grown in LB at 37°C with shaking of strains 50-wt, 50–64 and 50-rev were diluted to a similar OD (approximately 0.950 at 620 nm). A 1/100 dilution in fresh LB broth followed and bacterial growth was allowed at 37°C with shaking (540 rpm) in sterile 96-well microplates and assessed in an iEMS Multiskan Reader MF (Thermo Fisher Scientific). OD at 620 nm was determined every 15 minutes for 24 hours. Four independent assays were performed for each strain and standard deviation agreed to within 10%.

### Cell Envelope Protein Gel Electrophoresis

Bacterial pellets were harvested by centrifugation from 1.5 mL of an overnight culture grown in LB at 37°C with shaking. Pellets were rinsed twice with chilled Tris-Mg buffer (10 mM Tris-HCl, 5 mM MgCl_2_, pH 7.3) and finally resuspended in 1 mL of the same chilled buffer for sonication (5 cycles of 1 min of sonication followed by 1 min of rest) (Branson Sonifier 250). These samples were centrifuged for 2 min at 5,000 rpm, the supernatant was recovered and centrifuged again at 13,000 rpm for 30 min. Pellets were finally frozen.

A 12% SDS-polyacrylamide gel electrophoresis was run with the pellets resuspended in 1x Laemmli buffer. Gel was stained with Silver Staining Kit, Protein (GE Healthcare, Uppsala, Sweden). In order to characterize the protein bands of interest, they were recovered and sent to the Parc Cientific of Barcelona (Barcelona, Spain), where proteins were digested and sequenced through MALDI-TOF-TOF analysis.

### Adherence and Invasion Assays

Adherence and invasion assays were performed as previously described [49]. Briefly, monolayers of HeLa Ohio cells (ECACC 84211901) were grown by seeding 35 mm diameter tissue culture dishes (Corning, Corning, NY) with 5×10^5^ cells. Plates were incubated for 24 hours in minimum essential medium (MEM) (Gibco, Cergy Pontoise, France) supplemented with 10% fetal bovine serum (Gibco, Cergy Pontoise, France), 1% non-essential amino acids and 1/100 dilution of penicillin-streptomycin (10000 units-10 mg/mL) (Gibco, Cergy Pontoise, France) in a 5% CO_2_ atmosphere at 37°C, until a 55% confluency was reached. Cells were washed three times with MEM and fresh media was added with heat inactivated fetal bovine serum without antibiotics. Fresh overnight bacterial cultures incubated in LB at 37°C without shaking were used to infect each plate at a multiplicity of infection of 100. Plates were incubated for 2.5 h at 37°C with 5% CO_2_. For the adherence assay, infected monolayers were washed, fixed, stained with Giemsa and observed under a light microscope [49]. For the invasion assay, the infected monolayers were washed 3 times with MEM, fresh complete media containing gentamicin (100 µg/mL) was added and incubation for an additional 2 h was performed to kill extracellular bacteria. Monolayers were then washed 3 times with MEM and 1 mL of cold sterile water was added to lyse cells for 30 min at 4°C. Samples were pipetted vigorously and removed, diluted and plated on LB agar plates to determine the number of CFU (colony forming units) per monolayer. All experiments were performed at least three independent times and were carried out in duplicate.

### Microarray Analyses

Fresh cultures were inoculated in 15 mL LB with a 1/100 dilution of an overnight culture grown in LB at 37°C with shaking, and grown until strains reached the same OD_600_ values, between 0.5–0.6. Three mL were then taken and treated with 6 mL of RNAprotect Bacteria Reagent (Qiagen, Hilden, Germany). Mixtures were processed according to the manufacturer's instructions. Pellets were resuspended in 200 µL of TE buffer (10 mM Tris-Cl, 1 mM EDTA and pH 8.0) supplemented with 3 mg/mL lysozyme and vortexed, followed by an incubation at 32°C for 10 min with shaking. The RNA extraction was performed using RNeasy Mini Kit (Qiagen, Hilden, Germany) following the manufacturer's recommendations.

Three independent RNA samples of each strain were sent to the Unidad de Genómica of the Centro Nacional de Biotecnologia (Madrid, Spain) and processed according to previously described [50]. Briefly, a 70-mer oligonucleotide microarray constructed using the genome sequence of *S.* Typhimurium strain SL1344 was used for hybridization with the cDNA of each strain. Two separate experiments were performed. A normalized relative Cy5/Cy3 ratio >2 was considered as a significant increase in expression and a normalized relative Cy3/Cy5 ratio >2 was considered as a significant decrease in expression.

### RT-PCR

An aliquot of each of the same mRNA extractions used for microarray analyses was subsequently treated with DNA-free DNase (Ambion, Austin, TX, USA) according to the manufacturer's recommendations until RNA samples were totally DNA-free when checked by PCR using *gapA* (a housekeeping gene) primers. RT-PCR was performed using the AccessQuick RT-PCR System (Promega, Madison, WI, USA) and the primers listed in Table 5. The retrotranscription process was performed using 500 ng of RNA at 45°C for 45 min followed by a normal PCR program (as previously described), changing the number of cycles for each amplification as necessary. The annealing temperature and the number of cycles are detailed in Table 5. Samples were loaded in a GeneGel Excel (GE Healthcare, Uppsala, Sweden) at 600 V, 25 mA and 15 W for 1.5 h. Gel was stained with a DNA silver staining kit (GE Healthcare, Uppsala, Sweeden) according to the manufacturer's recommendations. Results were corroborated from two independent mRNA extractions and amplifications.

### Western Blotting

Bacterial strains were grown overnight in 50 mL LB at 37°C with shaking and were harvested by centrifugation. The pellet was rinsed twice with 10 mM Tris supplemented with 1% NaCl and was resuspended in 3 mL of the same buffer. Bacterial samples were sonicated on ice on a Vibra-Cell VCX 130 (Sonics) for a total of 3 min (30 s each cycle of sonication followed by 30 s of rest) with an amplitude of 50%. Cell debris were removed by centrifugation for 20 min at 4°C and 3500 rpm whereas the supernatant was collected and centrifuged again for 90 min at 4°C and 16000 rpm. The final pellet was resuspended in 1x PBS (Roche, Mannheim, Germany). Protein quantification was performed using the RC DC Protein Assay kit (Bio-Rad, Hercules, CA, USA) following the manufacturer's indications.

Ten µg of each protein sample were loaded in an 8% SDS-PAGE (Mini Protean II). Transference from gel onto a nitrocellulose membrane was performed for 2 h at 60 V on ice. The membranes were blocked using 1x PBS containing Tween 20 diluted 1/2000 (PBS-T) and 5% skim milk for 1 h at RT, followed by an overnight incubation at 4°C with the primary antibodies against AcrB and TolC proteins (Antibody Bcn, Barcelona, Spain) diluted 1/500 into PBS-T. The membranes were washed 3 times with PBS-T and once with PBS before secondary antibody, anti-rabbit IgG (GE Healthcare, Buckinghamshire, UK), diluted 1/2000 in PBS-T, was added for 1 h incubation at RT. The membranes were washed as previously described and processed using EZ-ECL (Biological Industries, Kibbutz Beit Haemek, Israel) for chemiluminescence detection of bands in a Fuji LAS-3000 equipment.

### Statistical Analysis

Differences in bacterial growth rate and percentage of invasion were assessed for significance by using Student's *t*-test (Statistical Package for the Social Sciences, SPSS 18.0). *P* values less than 0.05 were considered statistically significant at the 95% confidence interval.

## Supporting Information

Table S1 Includes additional data concerning microarray analysis
(0.20 MB DOC)
