# Supplementary material for: Repression of Invasion Genes and Decreased Invasion in a High-Level Fluoroquinolone-Resistant Salmonella Typhimurium Mutant
Source: PLoS One. 2009 Nov 25;4(11):e8029. doi: 10.1371/journal.pone.0008029 (PMC2777507; doi:10.1371/journal.pone.0008029)
Supplement: Table S1 — Includes additional data concerning microarray analysis (0.20 MB DOC) [file pone.0008029.s001.doc]

**Table S1. Complete report of microarrays results relative to the invasion phenotype.**

|  | |  |  | Microarray analyses^a,b^ | | | |
| --- | --- | --- | --- | --- | --- | --- | --- |
| Phenotype and gene | | Product |  | 50-64 vs 50-wt | | 50-rev vs 50-64 | |
| SPI-1 | invH | needle complex outer membrane lipoprotein precursor | | -4.32 | -2.33 | 1.74 | 1.34 |
|  | invF | invasion regulatory protein | | -8.04 | -4.42 | 1.41 | 1.12 |
|  | invG | outer membrane secretin precursor | | -6.10 | -3.49 | 1.14 | 1.53 |
|  | invE | invasion protein | | -4.77 | -3.02 | -1.08 | 1.02 |
|  | invA | needle complex export protein | | -2.28 | -2.23 | 1.47 | 1.06 |
|  | invB | secretion chaperone | | -11.70 | -6.40 | 2.99 | 2.49 |
|  | invC | type III secretion system ATPase | | -7.70 | -2.54 | 1.36 | 1.49 |
|  | invI | needle complex assembly protein | | -21.45 | -17.40 | 2.96 | 2.66 |
|  | invJ | needle length control protein | | -26.32 | -16.57 | 3.09 | 2.94 |
|  | spaO | type III secretion protein | | -6.12 | -3.26 | 1.13 | 1.56 |
|  | spaP | needle complex export protein | | -6.84 | -2.52 | 1.23 | 1.39 |
|  | spaQ | needle complex export protein | | -3.55 | -1.99 | 1.70 | 1.24 |
|  | spaR | needle complex export protein | | -1.02 | 1.20 | 1.26 | 1.07 |
|  | spaS | type III secretion protein | | -7.64 | -4.49 | 2.44 | 1.68 |
|  | sicA | secretion chaperone | | -15.45 | -14.35 | 4.74 | 3.92 |
|  | sipB | translocation machinery component | | -5.29 | -3.64 | 2.43 | 2.62 |
|  | sipC | translocation machinery component | | -3.52 | -2.27 | 2.55 | 2.15 |
|  | sipD | translocation machinery component | | -16.14 | -8.34 | 2.93 | 3.86 |
|  | sipA | secreted effector protein | | 4.74 | -2.06 | 1.76 | 2.96 |
|  | iacP | acyl carrier protein | | -5.39 | -2.60 | 3.21 | 2.90 |
|  | sicP | secretion chaperone | | -14.23 | -11.87 | 3.73 | 1.71 |
|  | sptP | protein tyrosine phosphatase/GTPase activating protein | | -4.50 | -2.88 | 2.13 | 1.70 |
|  | iagB | invasion protein precursor | | -8.29 | -2.97 | 1.98 | 1.80 |
|  | hilA | invasion protein transcriptional activator | | -8.54 | -4.88 | 1.95 | 1.57 |
|  | hilD | invasion protein regulatory protein | | -2.42 | -1.25 | 1.22 | 1.19 |
|  | prgH | needle complex inner membrane protein | | -3.36 | -3.03 | 2.97 | 1.68 |
|  | prgI | needle complex major subunit | | -1.09 | 1.02 | 1.28 | 1.10 |
|  | prgJ | needle complex minor subunit | | -3.21 | -2.08 | 2.02 | 2.09 |
|  | prgK | needle complex inner membrane lipoprotein | | -7.11 | -6.62 | 3.50 | 2.02 |
|  | orgA | needle complex assembly protein | | -4.98 | -2.93 | 2.11 | 2.13 |
|  | orgB | needlex complex export protein | | -11.26 | -5.86 | 3.91 | 2.12 |
|  | orgC | putative cytoplasmic protein | | -12.52 | -6.48 | 2.52 | 2.38 |
|  | hilC | invasion regulatory protein | | -5.51 | -3.01 | 1.89 | 1.63 |
|  | sprB | transcriptional regulator | | -10.41 | -5.70 | 2.41 | 1.53 |
|  | avrA | secreted effector protein | | -10.68 | -8.54 | 1.85 | 1.37 |
|  | sitD | putative permease | | -10.13 | -4.33 | 1.24 | 1.17 |
| Flagella |  |  |  |  |  |  |  |
|  | flgL | flagellar hook-associated protein | | -2.04 | -1.42 | 1.51 | 1.06 |
|  | flgK | flagellar hook-associated protein | | -3.92 | -2.19 | 2.27 | 2.02 |
|  | flgJ | flagellar biosynthesis protein | | -3.53 | -2.44 | 2.66 | 2.00 |
|  | flgI | flagellar P-ring protein precursor | | -2.82 | -2.67 | 1.71 | 1.47 |
|  | flgH | flagellar L-ring protein precursor | | -10.58 | -8.97 | 3.70 | 2.88 |
|  | flgG | flagellar basal-body rod protein | | -4.77 | -4.54 | 2.14 | 2.04 |
|  | flgF | cell-proximal portion of basal-body rod | | -11.74 | -5.36 | 3.12 | 2.85 |
|  | flgE | flagellar hook protein | | -2.96 | -1.86 | 3.09 | 2.51 |
|  | flgD | flagellar basal body rod modification protein | | -3.87 | -2.27 | 3.15 | 3.00 |
|  | flgC | flagellar basal body rod protein | | -6.10 | -5.10 | 3.82 | 3.49 |
|  | flgB | flagellar basal body rod protein | | -2.74 | -2.37 | 2.32 | 1.97 |
|  | flgA | flagellar basal body P-ring biosynthesis protein | | -2.64 | -2.15 | 1.37 | 1.04 |
|  | flgM | anti-FliA factor | | -1.79 | -1.41 | 1.43 | 1.24 |
|  | flgN | putative FlgK/FlgL export chaperone | | -1.44 | -1.33 | 1.18 | 1.36 |
|  |  |  |  |  |  |  |  |
|  | fljB | flagellar biosynthesis protein | | -3.01 | -1.93 | 4.11 | 4.89 |
|  | fljA | phase-1 flagellin repressor | | -2.25 | -1.65 | 3.98 | 2.82 |
|  |  |  |  |  |  |  |  |
|  | fliR | flagellar biosynthesis protein | | -1.19 | -1.22 | 1.12 | 1.39 |
|  | fliQ | flagellar biosynthesis protein | | -1.74 | -1.53 | 1.13 | 1.05 |
|  | fliP | flagellar biosynthesis protein | | -1.77 | -1.94 | 1.25 | 1.04 |
|  | fliO | flagellar biosynthetic protein | | -2.98 | -2.51 | 1.47 | 1.86 |
|  | fliN | flagellar motor switch protein | | -3.23 | -2.08 | 1.50 | 2.27 |
|  | fliM | flagellar motor switch protein | | -1.15 | -1.02 | -1.40 | 1.09 |
|  | fliL | flagellar biosynthesis protein | | -1.11 | -1.13 | -1.09 | 1.06 |
|  | fliK | flagellar hook-length control protein | | -1.27 | -1.35 | 1.48 | 1.46 |
|  | fliJ | flagellar protein | | -1.16 | 1.00 | -1.15 | 1.09 |
|  | fliI | flagellum-specific ATP synthase | | -3.23 | -2.02 | 1.66 | 1.61 |
|  | fliH | flagellar assembly protein | | -3.11 | -1.65 | 1.40 | 1.84 |
|  | fliG | flagellar motor protein | | -1.59 | -1.23 | 1.18 | 1.57 |
|  | fliF | flagellar M-ring protein | | -4.58 | -1.81 | 1.89 | 2.45 |
|  | fliE | flagellar basal body protein | | -2.29 | -1.97 | 1.25 | 1.40 |
|  | fliT | possible FliD export chaperone | | -2.78 | -2.60 | 1.87 | 2.47 |
|  | fliS | flagellar protein FliS | | -3.48 | -2.87 | 2.29 | 2.01 |
|  | fliD | flagellar hook-associated protein | | -1.55 | -1.42 | 1.09 | 1.19 |
|  | fliC | flagellar biosynthesis protein | | 1.05 | 1.02 | 1.20 | 1.19 |
|  | fliB | lysine-N-methylase | | -2.09 | -2.08 | -1.03 | 1.27 |
|  | fliA | flagellar biosynthesis sigma factor FliA | | -2.81 | -2.28 | 1.33 | 1.54 |
|  | fliZ | putative FliA-regulator | | -1.63 | -1.03 | 1.07 | 1.44 |
|  | fliY | putative periplasmic binding transport protein | | -1.96 | -1.57 | 1.00 | 1.63 |
|  |  |  |  |  |  |  |  |
|  | motA | flagellar motor protein | | -1.21 | -1.65 | 1.78 | 1.32 |
|  | motB | flagellar motor protein | | -1.83 | -2.26 | 1.74 | 1.79 |
|  |  |  |  |  |  |  |  |
|  | cheA | chemotaxis sensory histidine protein kinase | | 1.03 | -1.24 | -1.60 | 1.07 |
|  | cheW | chemotaxis docking protein | | -3.41 | -2.23 | 1.64 | 2.19 |
|  | cheM | methyl accepting chemotaxis protein II | | -1.87 | -1.61 | 1.47 | 1.67 |
|  | cheR | glutamate methyltransferase | | -1.75 | -1.42 | 1.58 | 1.67 |
|  | cheB | chemotaxis-specific methylesterase | | -3.91 | -3.36 | 2.20 | 2.14 |
|  | cheY | chemotaxis regulator | | -1.70 | -1.13 | 1.88 | 1.54 |
|  | cheZ | chemotactic response protein | | -2.13 | -1.49 | 1.27 | 1.72 |

^a^ each microarray analysis is provided with two independent data

^b^ + indicates up-regulation of the genes, – indicates down-regulation

^c^ gene expression of 50.64 relative to expression of 50.wt

^d^ gene expression of 50.rev relative to expression of 50.64
